# Low‐dose vemurafenib in hairy cell leukemia patients with active infection

**DOI:** 10.1002/ajh.25474

**Published:** 2019-04-04

**Authors:** Jan‐Paul Bohn, Andreas Pircher, David Wanner, David Vill, Bernhard Foeger, Dominik Wolf, Michael Steurer

**Affiliations:** ^1^ Internal Medicine V, Hematology and Oncology Medical University of Innsbruck Innsbruck Austria; ^2^ Department of Internal Medicine Academic Teaching Hospital Hall Hall Austria; ^3^ Department of Internal Medicine Hospital Bregenz Bregenz Austria; ^4^ Medical Clinic 3 University Hospital Bonn Bonn Germany

*To the Editor*:

Classic hairy cell leukemia (HCL) is a rare indolent B‐cell malignancy.^1^ Standard treatment with purine analogues is highly effective, but also associated with profound myelotoxicity.^2^, ^3^ As such, patients with ongoing infection do not qualify for purine analogues and still represent a therapeutic challenge. Recently, the BRAF‐inhibitor vemurafenib was suggested an adequate treatment alternative,^4^ but feasibility data remain scarce.

We here report our long‐term experience of six HCL patients with active infection, who achieved durable hematological remissions with low‐dose vemurafenib.

**Case 1**: A 68‐year‐old woman was diagnosed with HCL 14 years ago. Frontline treatment with cladribine achieved a complete remission (CR) of 104 months. With 84% hairy cell (HC) infiltration in the bone marrow (BM) at relapse re‐/treatment with cladribine and rituximab did not yield any response, but resulted in severe infectious complications (Table S1). In the absence of other approved therapeutic alternatives, vemurafenib (480 mg/day) was applied off‐label with confirmed BRAF V600E mutation. Within 2 weeks rapid recovery of peripheral blood (PB) counts (Figure 1) and adequate infection control was achieved. Treatment was well tolerated without any adverse events (AE). Due to excellent response and risk of secondary skin cancers, vemurafenib was discontinued after 3 months and BM biopsy confirmed CR (ongoing for 76 months).

**Figure 1:**
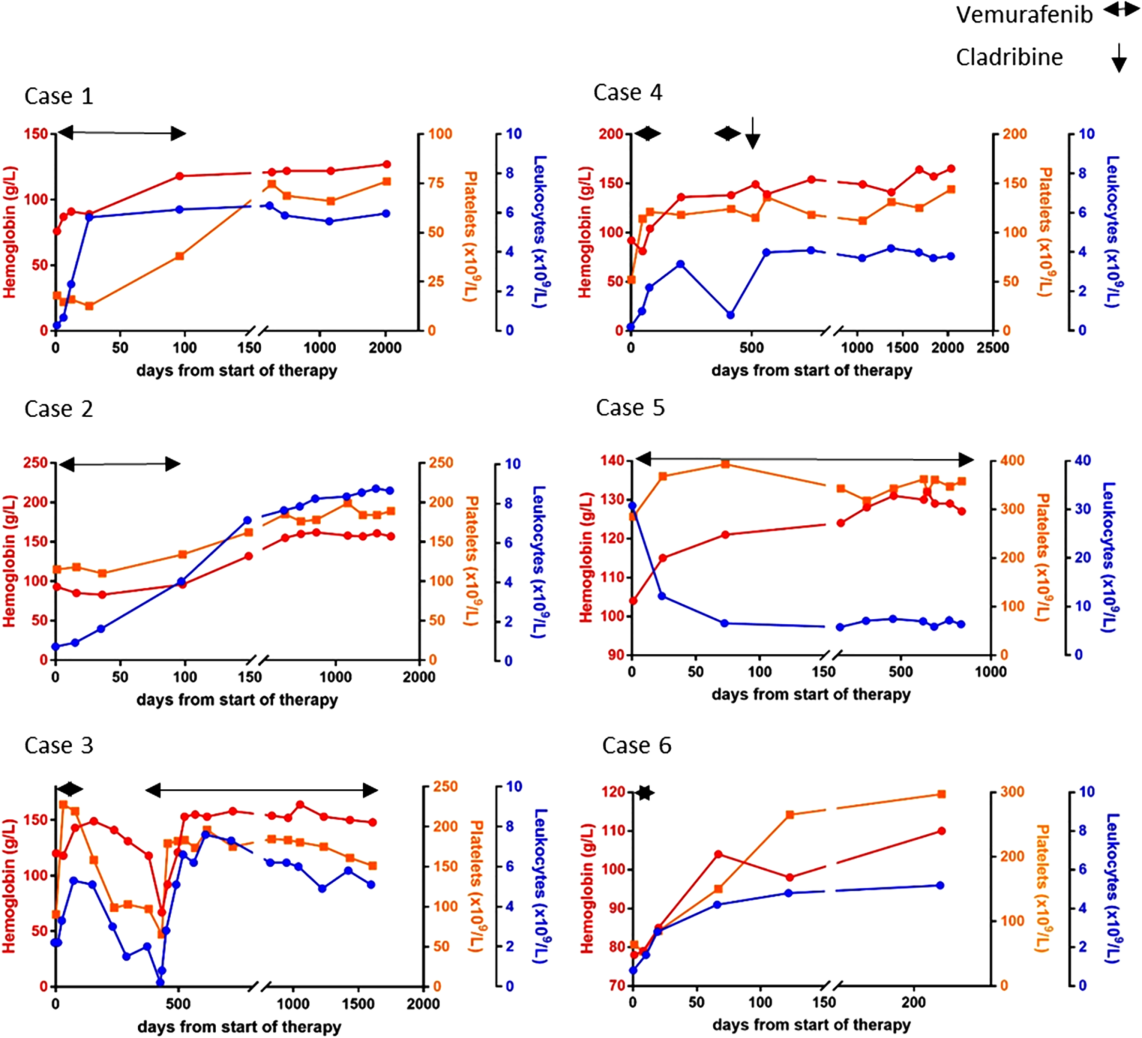
Hematologic activity of low‐dose vemurafenib in our HCL patients. HCL, hairy cell leukemia [Color figure can be viewed at wileyonlinelibrary.com]

**Case 2**: Presenting with bilateral pneumonia and *Pseudomonas aeruginosa* bacteremia, a 70‐year‐old male was diagnosed with BRAF V600E positive HCL (75% HC infiltration in BM) in 06/2013. Minimizing additional myelotoxicity, frontline treatment with rituximab was applied (Table S1). In the absence of any objective response and progressive bipulmonary infiltrates, off‐label treatment with vemurafenib (960 mg/day) was initiated. Leukocyte counts started to recover within 2 weeks (Figure 1) allowing resolution of infection. No AE were noted. Vemurafenib was stopped after 3 months due to concerns about secondary skin tumors and BM biopsy confirmed CR (ongoing for 63 months).

**Case 3**: A now 71‐year‐old man was diagnosed with HCL 23 years ago. Repeated courses of cladribine and rituximab achieved a progression‐free survival (PFS) of 93 months, but eventually the patient presented with refractory disease (65% HC infiltration in BM, Table S1). At relapse complicated by pneumonia off‐label salvage treatment with vemurafenib (480 mg/day) resulted in rapid amelioration of leukocyte counts within 2 weeks (Figure 1) and resolution of infection. Given the risk of secondary skin malignancies, vemurafenib was ended in partial remission (PR) after 3 months. At relapse 5 months later, retreatment achieved rapid PR again (Figure 1). Due to excellent tolerability, limited PFS and PR as deepest response, now vemurafenib has been given continuously (41 months) serial dermatologic surveillance provided.

**Case 4**: A 68‐year‐old male HCL patient (nearly 100% HC infiltration in the BM, BRAF V600E mutated) was referred to our clinic with grade 4 neutropenia and invasive pulmonary aspergillosis in 10/2012. Avoiding additional myelotoxicity, off‐label treatment with vemurafenib (960 mg/day) was applied. Within 5 weeks leukocyte counts started to recover (Figure 1) allowing resolution of pulmonary aspergillosis. Vemurafenib was discontinued at month 3 in PR (BM biopsy: <40% HC infiltration) owing to concerns about secondary skin cancers. At relapse 10 months later vemurafenib (480 mg/day) re‐exposure was chosen due to pneumonia. PR was achieved after 3 months now allowing cladribine consolidation therapy (Table S1).

**Case 5**: A now 82‐year‐old woman was diagnosed with HCL at the age of 47 years. Repeated courses of interferon‐α, cladribine, and pentostatin achieved a CR of over 27 years, but also induced prolonged grade 3/4 neutropenia (Table S1). At relapse complicated by recurrent urinary tract infections vemurafenib (480 mg/day) was given as off‐label treatment with confirmed BRAF V600E mutation. Within a few weeks HC were cleared from PB (Figure 1). Given PR as deepest response 3 months on treatment (0.5% HC in PB) and excellent tolerability, vemurafenib has been applied continuously for 32 months so far. Serial dermatologic follow‐up included, no AE have been noted.

**Case 6:** A 69‐year‐old male patient was transferred to our intensive care unit with septic shock and multi‐organ failure despite broadest antibiotic, antifungal, and antiviral therapy in 12/2017. Grade 4 pancytopenia was initially interpreted as infection‐associated, but BM examination revealed 70% BRAF V600E mutated HC infiltration. Vemurafenib (480 mg/day) was started as off‐label salvage treatment. After 2 weeks, amelioration of leukocyte counts (Figure 1) favored control of life‐threatening infection and restoration of lung, kidney, and liver function. On day 14 vemurafenib was prematurely discontinued based on transient liver enzyme elevation. Nevertheless, the patient remains in PR with a follow‐up of 10 months.

Given an overall response rate of nearly 100% without additional myelotoxicity in 60 relapsed/refractory HCL patients, the BRAF‐inhibitor vemurafenib (1920 mg/day) promised to offer an ideal salvage treatment for those with active infection.^4^, ^5^ Study enrollment criteria, however, excluded this patient subpopulation and the feasibility of vemurafenib in this setting remains to be better defined.

Moreover, optimal vemurafenib dosing and scheduling in HCL is still unclear as AE required dose reduction in >50% of study patients^4^ and lower doses (480‐960 mg/day) seem similarly effective.^6^ Finally, other than in melanoma in HCL trials treatment‐time was limited to a few months due to concerns about drug‐related skin tumors, partially contributing to a median PFS of only approximately 9 months.^4^, ^5^

We here describe six HCL patients with active infection in which any additional myelotoxicity had to be avoided. Off‐label salvage treatment with a short course of low‐dose vemurafenib (480‐960 mg/day) achieved rapid and, mostly, durable hematological responses allowing resolution of infection in all patients. Treatment was very well tolerated and none of the patients required dose modification.

All relapsed patients remained drug‐sensitive. However, in HCL quality of responses achievable with vemurafenib frequently declines with each successive retreatment, making intermittent dosing seem unrewarding in patients with generally short PFS and high infection risk at relapse.^4^ In these patients our long‐term follow‐up illustrates feasibility and efficacy of continuous vemurafenib dosing until disease progression as done in melanoma. In patients not relapsed/refractory to purine analogues +/− rituximab our data suggest that rapid infection control with a short course of vemurafenib may provide if necessary a window of opportunity to subsequent consolidation therapy.

## CONFLICT OF INTEREST

The authors declare no competing interests.

## Supporting information


**Table S1** Clinical outcome of consecutive treatments in our HCL patients
