# Supplementary material for: Low‐dose vemurafenib in hairy cell leukemia patients with active infection
Source: Am J Hematol. 2019 Apr 4;94(6):E180–2. doi: 10.1002/ajh.25474 (PMC6593695; doi:10.1002/ajh.25474)
Supplement: Supplementary file 1 — Table S1 Clinical outcome of consecutive treatments in our HCL patients [file AJH-94-E180-s001.docx]

| **Table 1** Clinical outcome of consecutive treatments in our HCL patients | | | | | | | |  |
| --- | --- | --- | --- | --- | --- | --- | --- | --- |
| Case 1 | | | | | | | |  |
| Line of treatment | Regimen | Dosage | Date | Treatment duration | Response | Progression-free survival | Grade III/IV toxicity |  |
| 1 | Cladribine | 0.14 mg/kg/day | 11/2002 | 5 days (1 cycle) | CR | 104 months | Pancytopenia |  |
| 2 | Rituximab | 375 mg/m² | 07/2011 | Weekly x 4 | None | - | Recurrent infectious complications due to prolonged neutropenia |  |
| 3 | Cladribine | 0.14mg/kg/day | 01/2012 | 5 days (1 cycle) | None | - | Pneumonia, urosepsis with septic shock |  |
| 4 | Vemurafenib | 480 mg/day | 07/2012-10/2012 | 3 months | CR | 76 months (ongoing) | None |  |
| Case 2 |  |  |  |  |  |  |  |  |
| Line of treatment | Regimen | Dosage | Date | Treatment duration | Response | Progression-free survival | Grade III/IV toxicity |  |
| 1 | Rituximab | 375 mg/m² | 07/2013 | Weekly x 4 | None | - | Pneumonia due to prolonged neutropenia |  |
| 2 | Vemurafenib | 480 mg/day | 08/2013-11/2013 | 3 months | CR | 63 months (ongoing) | None |  |
| Case 3 |  |  |  |  |  |  |  |  |
| Line of treatment | Regimen | Dosage | Date | Treatment duration | Response | Progression-free survival | Grade III/IV toxicity |  |
| 1 | Cladribine | 0.14 mg/kg/day | 10/1993 | 5 days (1 cycle) | PR | 22 months | Neutropenia |  |
| 2 | Cladribine | 0.14 mg/kg/day | 08/1995 | 5 days (1 cycle) | PR | 24 months | Febrile neutropenia |  |
| 3 | Cladribine | 0.14 mg/kg/day | 10/2003 | 5 days (1 cycle) | PR | 19 months | Neutropenia, thrombopenia |  |
| 4 | Cladribine | 0.14 mg/kg/day | 03/2011 | 5 days (1 cycle) | None | - | Neutropenia, thrombopenia |  |
| 5 | Rituximab | 375 mg/m² | 06/2011 | Weekly x 4 | PR | 28 months | None |  |
| 6 | Rituximab | 375 mg/m² | 10/2013 | Weekly x 4 | None | - | Pneumonia due to prolonged neutropenia |  |
| 7 | Vemurafenib | 480 mg/day | 03/2014 | 3 months | PR | 8 months | None |  |
| 8 | Vemurafenib | 480 mg/day | 11/2014 | continuously | PR | 41 months (ongoing) | None |  |
| Case 4 |  |  |  |  |  |  |  |  |
| Line of treatment | Regimen | Dosage | Date | Treatment duration | Response | Progression-free survival | Grade III/IV toxicity |  |
| 1 | Vemurafenib | 960 mg/day | 11/2012 – 03/2013 | 5 months | PR | 10 months | None |  |
| 2 | Vemurafenib | 480 mg/day | 09/2013 – 12/2013 | 3 months | PR |  | None |  |
| 3 | Cladribine | 0.14 mg/kg/day | 04/2014 | 5 days (1 cycle) | PR | 55 months (ongoing) | Neutropenia |  |
| Case 5 |  |  |  |  |  |  |  |  |
| Line of treatment | Regimen | Dosage | Date | Treatment duration | Response | Progression-free survival | Grade III/IV toxicity |  |
| 1 | splenectomy | - | 05/1982 | - | PR | 12 months | None |  |
| 2 | Interferon-α | 1.5 x 10^6^ U/week | 06/1983 – 06/1986 | 36 months | PR | 36 months | None |  |
| 3 | Pentostatin | 4 mg/m² | 07/1986 – 03/1987 | 8 months***** | CR | 163 months | Recurrent infectious complications due to prolonged neutropenia |  |
| 4 | Interferon-α | 4.5-9 x 10^6^ U/week | 01/2000-11/2007 | 95 months | PR | 95 months | None |  |
| 5 | Cladribine | 0.14 mg/kg/day | 11/2007 | 5 days (1 cycle) | CR | 49 months | Recurrent infectious complications due to prolonged neutropenia |  |
| 6 | Interferon-α | 4.5 x 10^6^ U/week | 12/2011-08/2014 | 33 months | PR | 33 months | None |  |
| 7 | Vemurafenib | 480 mg/day | 03/2016 | continuously | PR | 32 months (ongoing) | None |  |
| Case 6 |  |  |  |  |  |  |  |  |
| Line of treatment | Regimen | Dosage | Date | Treatment duration | Response | Progression-free survival | Grade III/IV toxicity |  |
| 1 | Vemurafenib | 480 mg/day | 01/2018 | 2 weeks | PR | 10 months (ongoing) | None |  |
| **CR, complete remission**. A complete remission was defined as the morphological absence of hairy cells in blood and bone marrow in combination with complete resolution of cytopenia and organomegaly. | | | | | | | | |
| **PR, partial remission**. A partial remission was defined as a complete resolution of cytopenia in combination with at least 50% decrease in organomegaly and hairy cell infiltration of the bone marrow. | | | | | | | | |

*****4 mg/m² per week x 3, then every other week x 3, then once monthly x 6.
